# The Outcome of Distal Radius Fractures with Concomitant Injuries Is Similar to those of Isolated Distal Radius Fractures Provided that an Arthroscopically Supported Treatment Is Performed

**DOI:** 10.3390/jcm9040974

**Published:** 2020-04-01

**Authors:** Francesca von Matthey, Karola Schmid, Michael Zyskowski, Stephan Deiler, Peter Biberthaler, Helen Vester

**Affiliations:** 1 Clinic for Traumatology, Klinikum rechts der Isar, Technical University of Munich, 81667 Munich, Germany; Francesca.von.matthey@tum.de (F.v.M.); karolaschmid@gmx.de (K.S.); Michael.Zyskowski@mri.tum.de (M.Z.); Peter.Biberthaler@mri.tum.de (P.B.); 2 Clinic for Traumatology, Department for Handsurgery, Klinikum rechts der Isar, Technical University of Munich, 81667 Munich, Germany; stephan.deiler@me.com

**Keywords:** distal radius fracture, wrist arthroscopy, TFCC lesion, scapholunate ligament rupture

## Abstract

Background: Concomitant injuries of distal radius fractures (DRF) can have a fatal impact on the patients’ outcome. However, wrist arthroscopy is a costly and complex procedure. It remains elusive whether patients benefit from an additional arthroscopy. Methods: Patients with a DRF who were treated arthroscopically were enrolled. Fifty-six wrists were evaluated regarding their function by self-assessment with the Munich Wrist Questionnaire (MWQ). Thirty-nine patients were examined for postoperative strength and motion. Concomitant injuries were detected. Results: A total of 75% of the DRF were type C injuries (AO classification). Twenty-four cases (43%) were triangular fibrocartilaginous complex (TFCC) lesion, eight cases (14%) of scapholunate ligament (SL) injuries and seven cases (12%) were a combination of TFCC and SL ligament lesion. No difference in function could be detected between DRF with surgically addressed concomitant lesions and isolated DRF. Dorsalextension, palmarflexion and grip strength were significantly reduced in patients with DRF and concomitant injuries compared to the healthy wrist. However, patients with DRF and arthroscopically treated concomitant injuries had similar results to those suffering only from an isolated DRF. Conclusion: The increased occurrence of concomitant injuries is to be expected in intraarticular DRF. Patients with concomitant injuries benefit from an arthroscopically assisted fracture treatment and show similar results compared to isolated DRF.

## 1. Introduction

Fractures of the distal radius are the most common human fractures and require a complex diagnostic and treatment strategy [1,2]. Aims of the treatment include the restoration of wrist function and the long-term absence of pain. Therefore, a trend towards an operative procedure has been shown within the recent years [1,3,4]. Numerous surgical options are available ranging from plate osteosynthesis over fixateur extern or K-wire osteosynthesis up to arthroscopic procedures. However, conservative treatment, especially in elderly patients with underlying health conditions, should be considered as well [5].

The detection of concomitant injuries, as well as the quality of the reconstruction, is decisive for the functional outcome. In complex distal radius fractures (DRF), concomitant injuries of the triangular fibrocartilaginous complex (TFCC) and the scapholunate ligament (SL ligament) have been reported in more than 30–70% of cases [6,7]. Unrecognized accompanying injuries can lead to persistent discomfort and impaired function in the wrist even after adequate fracture treatment [1,8]. In recent years, it has been shown repeatedly that the total extent of the injury in DRF can be determined only in the context of wrist arthroscopy. Preoperative imaging alone is often insufficient to adequately assess the severity of the accompanying injuries [1,8,9,10,11]. The statements in the literature on the indication of an arthroscopic fracture supply diverge. This is how Deiler et.al, based on the current literature, have developed a treatment algorithm and recommend the preparation of computed tomography in the presence of a DRF AO type B or C. In the case of a joint level >1 mm, free articular bodies or impaction cavities, arthroscopic fracture treatment is recommended [1]. With the arthroscopically assisted fracture treatment, not only concomitant injuries can be identified and addressed, but it also contributes to the improvement of the intraoperative reduction result [12]. Hardly any data has yet been available to evaluate the functional outcome in patients with detected and simultaneously arthroscopically treated concomitant injuries in DRF. Moreover, it remains elusive whether the additional arthroscopy has enough benefit to be worth the effort. 

Therefore, the aim of this study was to detect the occurrence of accompanying injuries in distal radius fractures and to examine the functional outcome after an arthroscopically assisted fracture treatment. We hypothesized that patients with DRF and concomitant injuries that are treated arthroscopically benefit from arthroscopy and have comparable outcomes as patients suffering from an isolated DRF. 

## 2. Methods

### 2.1. Ethical Approval

All investigations were carried out following the Declaration of Helsinki of 1975, revised in 2013. The study was approved by the local ethics committee under reference no. 327/15s. All patients provided their informed consent prior to participation. 

### 2.2. Study Population

The present retrospective study included all patients who presented in our hospital during the period from July 2012 to February 2015 and underwent an arthroscopically assisted treatment of a fracture of the distal radius. The inclusion criteria were a radiographic confirmed distal radius fracture, which needed operative treatment because of displacement, instability or intraarticular fragments. An additional criterion was the indication for an arthroscopically assisted fracture treatment according to the published criteria of Deiler et al. [1], or a concomitant injury already confirmed by preoperative MRI imaging. Mean follow-up time after surgery was 3.4 ± 0.5 (mean ± SEM) years. Exclusion criteria were open fractures, polytrauma and conservatively treatable fractures.

### 2.3. Control Population

In the context of clinical follow-up of flexibility and strength, the healthy wrist and the wrist with an isolated distal radius fracture served as control.

### 2.4. Surgery

All surgical procedures were performed by two senior surgeons. Patients were operated either under general anesthesia or in plexus anesthesia. The fracture treatment was standardized in the patient’s supine position via standard volar access. The fracture supply was carried out by means of plate osteosynthesis (Aptus, Fa. Medartis).

**Figure 1:**
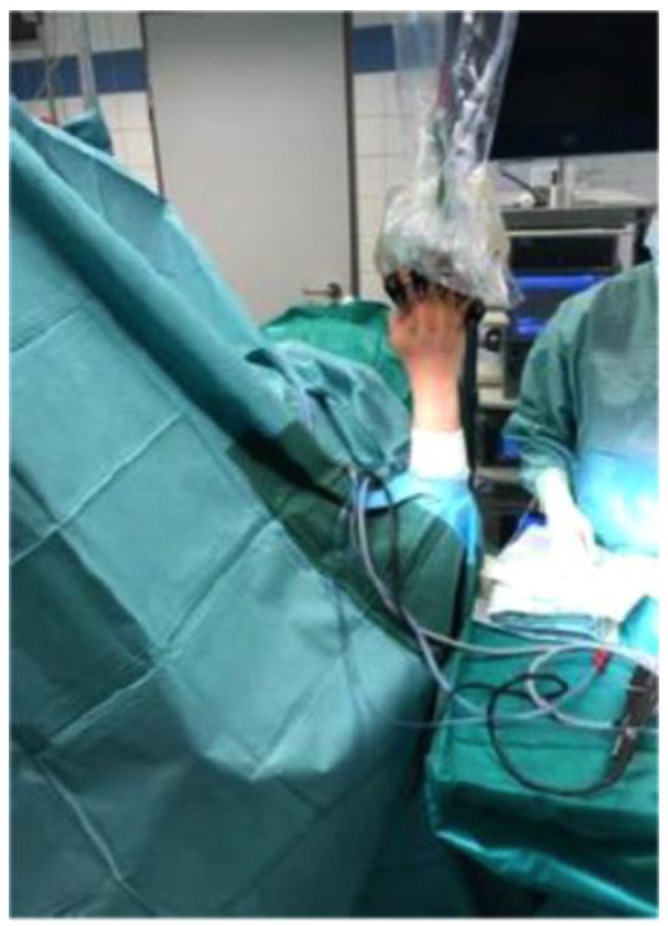
Construction arthroscopy.

For wrist arthroscopy, arthroscopy was performed on the affected wrist and accompanying injuries were addressed. For this purpose, the affected arm was abducted and flexed at 90° in the elbow joint and hung under a 2 kg extension using an arthroscopy plate. For arthroscopy, a 2.7 mm, 30° angle optic (Storz) was used. As arthroscopic portals, the dorsal standard portals were used in the sense of the dorsal 3/4 portal and the 6R portal [13] (Figure 1).

**Figure 2:**
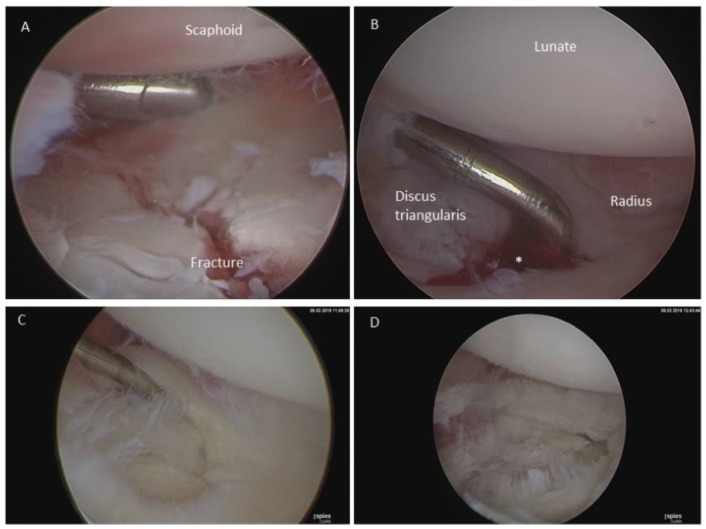
Arthroscopy. (**A**) Arthroscopically assisted distal radius fracture. The fracture has been set anatomically; however, the patient also has a radial discus rupture type 1D according to the Palmer classification, which is unstable, (**B**) and has to be repaired; (**C**) a central discus tear type 1A according to the Palmer classification; (**D**) discus after a stable resection of the ruptured part.

Arthroscopically, the fracture reduction was examined, as was the wrist, for accompanying injuries. In the case of an existing TFCC lesion, either a partial discus resection for the central lesions or a discus suture for the ulnar lesions was performed. SL ligament injuries were classified according to the Geissler classification [9]. All treated SL ligament ruptures of this collective were traumatic Geissler III and IV ruptures. In the presence of SL ligament injuries, a temporary carpal arthrodesis with K-wires occurred (Figure 2).

### 2.5. Primary Outcome Measures

For the present study a number of primary and secondary target parameters were investigated.

### 2.6. Demographic Target Parameters

Age, gender, fracture type according to AO classification and accompanying injuries were documented, as was the time interval between surgical treatment and follow-up.

### 2.7. Subjective Function Parameters

In this connection, according to the Patient Outcome Reported Measurements guidelines, the function of the wrist was examined with a validated questionnaire. The Munich Wrist Questionnaire [14] captures the relevant function of the wrist according to several well-established scoring systems, such as the DASH or SPADI score.

The MWQ is a validated self-assessment questionnaire, which addresses questions concerning pain, mobility and everyday function. Each item is rated with a set score. The maximum achievable score is 250 (100%) and corresponds to a full wrist function. 

### 2.8. Objective Target Parameters

For this purpose, the strength was measured in kilograms using a dynamometer (Saehan Corp., Masan, Korea). The range of motion was measured in angular degrees by the Neutral-0 method and expressed in mean ± SEM.

### 2.9. Statistical Analysis

The statistical analysis was performed by Sigmastat (Microsoft, Erkrath, Germany) according to the data quality descriptively or analytically by Mann–Whitney U test for ordinal distributed data or *t*-test for independent samples for normally distributed data. A paired *t*-test or a signed-rank test was performed to compare dependent data. The data was also described according to the data quality in (%) or as mean ± SEM. A level of significance as *p* < 0.05 was accepted.

## 3. Results

### 3.1. Study Population

**Figure 3:**
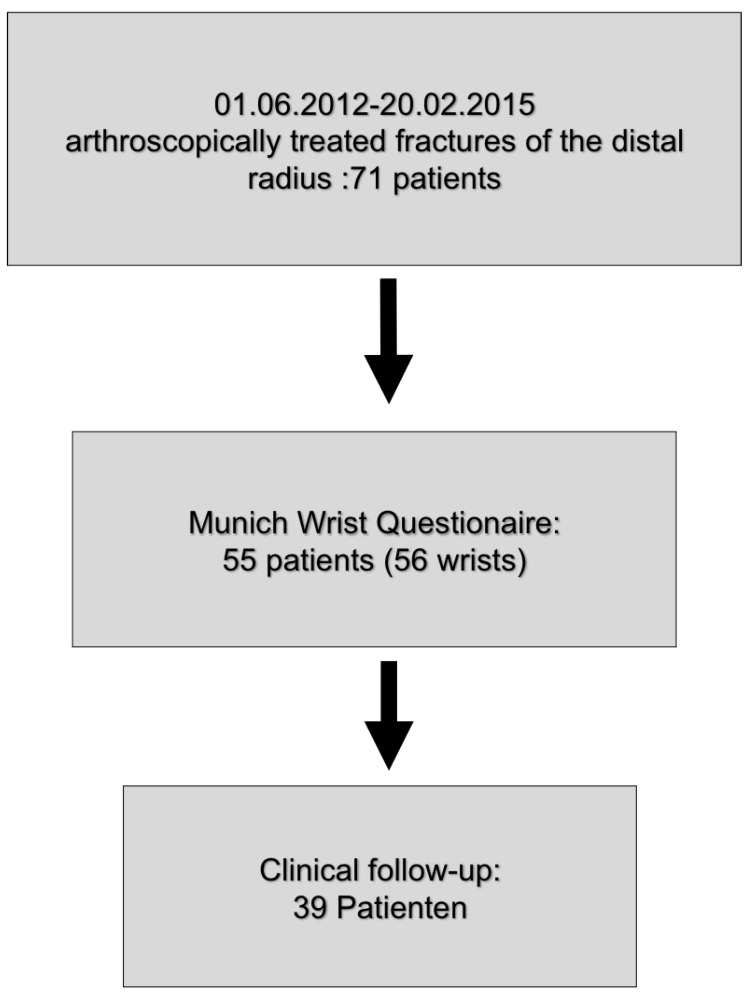
Study collective. In the period between June 2012 and February 2015, 71 wrists of 72 patients were operated on by arthroscopically assisted fracture treatment. Of these, 56 wrists from 55 patients were included in our study. In addition, 39 patients were examined clinically.

Between June 2012 and February 2015, 72 wrists from 71 patients underwent arthroscopically assisted fracture treatment. Of these, 56 wrists from 55 patients were included in our study. Unfortunately, the other patients could not be enrolled, as they did not reply to our letter of inquiry. Subjectively, this collective was followed up by means of MWQ. In addition, 39 wrists were clinically re-examined (Figure 3).

### 3.2. Demographic Target Parameters

**Figure 4:**
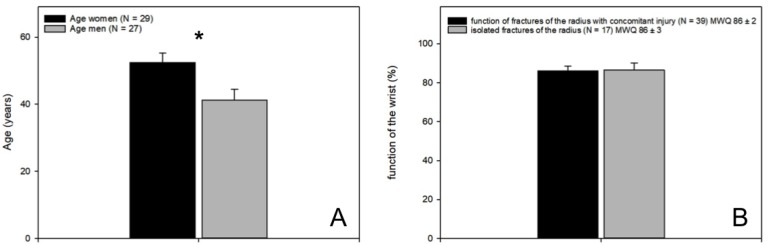
Age distribution and function. (**A**) The female collective was significantly older than the male patients; (**B**) no difference in function of the wrist could be detected according to the self-assessment score (MWQ) between fractures with arthroscopically treated concomitant injuries and isolated fractures of the distal radius (86% ± 2% vs. 86% ± 3%, mean ± SEM).

Of the 55 surgically treated patients (56 wrists), 27 were men and 29 were women. With a mean age of 41 ± 3 years of male patients and 52 ± 3 years of female patients, the women were significantly older (*p* = 0.005; Figure 4A).

Of all 56 wrists examined by MWQ, five were (9%) type A fractures, nine (16%) type B fractures and 42 cases (75%) were type C fractures. In 24 cases (43%), there was an isolated TFCC lesion as a concomitant injury, in eight cases (14%) an isolated injury of the SL ligament, and in seven cases (12%), a combination of TFCC lesion and lesion of the SL ligament occurred (Table 1). TFCC lesions were either arthroscopically sutured or a partial discus resection was performed according to the localization of the lesion. No open repair was needed, as the distal radioulnar joint was stable after arthroscopic fixation.

Five fractures were classified as type A fractures (9%), nine cases (16%) were type B fractures and 42 cases (75%) were type C fractures. In 24 cases (43%), there was an isolated TFCC lesion as a concomitant injury, in eight cases (14%) an isolated SL ligament injury, and in seven cases (12%), a combination of TFCC and SL ligament lesion.

The mean age of all patients (*n* = 55) was 47 years (47 ± 2). The mean age of type A fractures averaged 46 years (46 ± 9), type B fractures 34 (34 ± 4) years and type C fractures 50 (50 ± 2) years.

The patients with type B fractures were significantly younger (*p* < 0.05, *t*-test) than those with type C fractures. Patients with isolated injuries of the SL ligament were also significantly younger compared to those with isolated TFCC lesions (*p* = 0.043). Patients with isolated SL ligament lesions also showed a significantly younger collective compared to patients with a TFCC lesion in combination with a SL ligament lesion (*p* = 0.042).

### 3.3. Follow-Up by Self-Assessment Score (MWQ)

Of all 56 examined wrists, there was no difference concerning function between type A, type B and type C fractures. On average, fractures of the distal radius with surgically addressed concomitant lesions did not show a significant difference concerning their function (86% ± 2%) compared to fractures without any concomitant lesion (86% ± 3%; Figure 4B). Even between the individual accompanying injuries, no functional difference could be detected after surgical treatment of the injury (Table 2).

On average, the distal radius fractures with surgically addressed concomitant injuries showed no significant functional difference compared to fractures without accompanying injuries (both 86% ± 2% (mean ± SEM)). Even between individual accompanying injuries, no functional difference could be detected after surgical treatment of the injury.

### 3.4. Clinical Follow-Up

#### 3.4.1. Grip Strength

**Figure 5:**
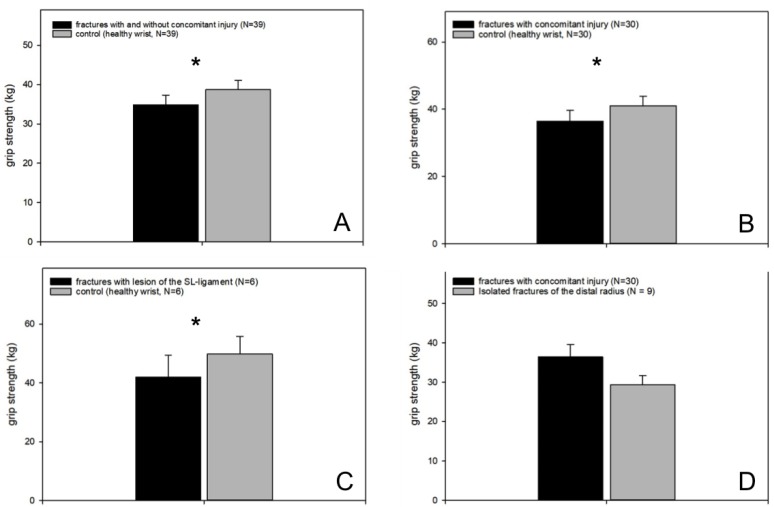
Grip strength. (**A**) Statistically significant reduced grip strength in the injured hand compared to the healthy wrist; (**B**) patients suffering from distal radius fractures with concomitant injuries had a significantly reduced grip strength compared to the healthy wrist; (**C**) patients suffering from distal radius fractures and SL ligament tears had a significantly reduced grip strength compared to the control; (**D**) no difference between patients suffering from isolated distal radius fractures and those with distal radius fractures and arthroscopically treated concomitant lesions.

In terms of strength, there has been a statistical significance between the injured wrist and the healthy opposite (*p* = 0.005, fractures with and without concomitant injuries vs. healthy wrist, *n* = 39, Wilcoxon signed-rank test; Figure 5 A). 

Specifically, we found a significant difference between distal radius fractures in combination with concomitant injuries compared to the healthy side (*p* = 0.01, fractures with concomitant injuries vs. healthy wrist, *n* = 30, Wilcoxon signed-rank test; Figure 5B). No difference was detected between isolated fractures of the distal radius and the healthy wrist (*p* = 0.27).

The more detailed data analysis showed a significant difference in the strength between distal radius fractures in combination with SL ligament lesions and the healthy wrist (*p* = 0.05, fractures wit SL ligament lesions vs. healthy wrist, *n* = 6, Wilcoxon signed-rank test; Figure 5C), whereas no difference between fractures in combination with TFCC lesions or TFCC lesions and SL ligament lesions could be found.

Patients with a distal radius fracture and arthroscopically treated concomitant injuries showed no significant difference concerning the grip strength compared to patients suffering from isolated distal radius fractures (Figure 5D). 

#### 3.4.2. Range of Motion

**Figure 6:**
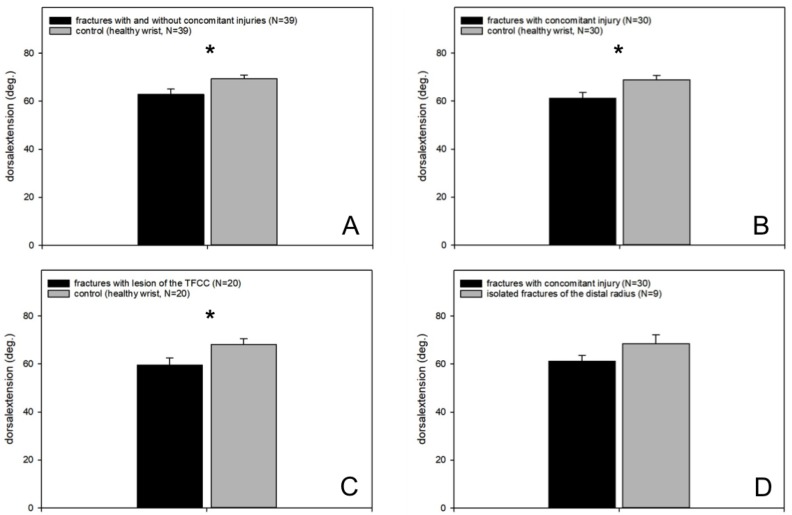
Dorsalextension. (**A**) Significantly reduced dorsalextension in the injured wrist (fracture of the distal radius with and without concomitant injury) compared to the healthy wrist; (**B**) significantly reduced dorsalextension in fractures with concomitant injuries compared to the healthy wrist; (**C**) significantly reduced dorsalextension in fractures with concomitant triangular fibrocartilaginous complex (TFCC) lesions compared to the healthy wrist; (**D**) no difference between patients suffering from isolated distal radius fractures and those with distal radius fractures and arthroscopically treated concomitant lesions.

However, all injured wrists regardless of possible concomitant injuries showed a significantly reduced dorsalextension compared to the healthy opposite wrist (*p* = 0.002, fractures with and without concomitant injuries vs. healthy wrist, *n* = 39, paired *t*-test; Figure 6A). 

In the more detailed analysis of the data we could find a significantly lower dorsalextension of distal radius fractures with concomitant injuries compared to the healthy wrist (*p* = 0.004, fractures with concomitant injuries vs. healthy wrist paired *t*-test, *n* = 30; Figure 6B), whereas the dorsal extension of isolated distal radius fractures compared to the healthy wrist was not significantly reduced (*p* = 0.2). 

The comparison of distal radius fractures in combination with a TFCC lesion showed a significantly reduced dorsalextension in the affected wrist (*p* = 0.008, fractures with lesions of the TFCC vs. healthy wrist, paired *t*-test, *n* = 20; Figure 6C), whereas fractures of the distal radius with isolated lesions of the SL ligament or a combination of TFCC and SL ligament lesion did not show a statistically significant difference (*p* = 0.3 or 0.7). No difference was found between patients with distal radius fracture in combination with a TFCC lesion and patients with distal radius fracture in combination with a lesion of the SL ligament (*p* = 0.36).

Patients with distal radius fractures and arthroscopically treated concomitant injuries showed no significant difference in dorsalextension compared to patients suffering from isolated distal radius fractures (Figure 6D). 

**Figure 7:**
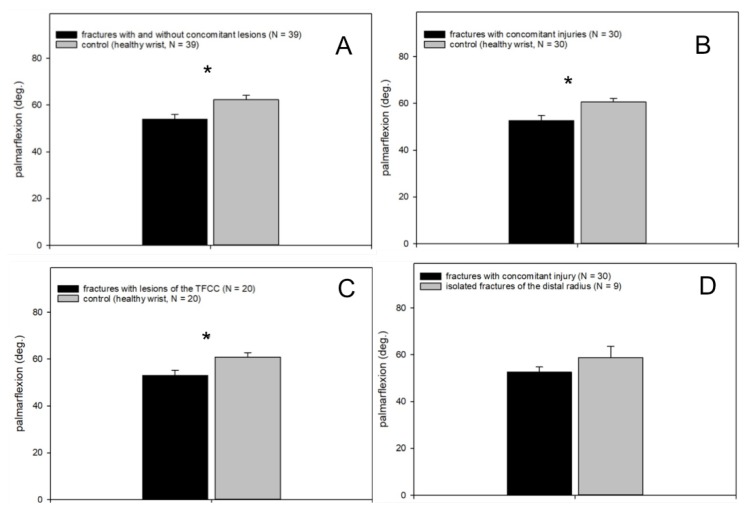
Palmarflexion. (**A**) Significantly reduced palmarflexion in the injured wrist (fracture of the distal radius with and without concomitant injuries) compared to the healthy wrist; (**B**) significantly reduced palmarflexion in fractures with concomitant injuries compared to the healthy wrist; (**C**) significantly reduced palmarflexion in fractures with concomitant TFCC lesions compared to the healthy wrist; (**D**) no difference between patients suffering from isolated distal radius fractures and those with distal radius fractures and arthroscopically treated concomitant lesions.

The analysis of palmarflexion was similar, with a significantly reduced range of motion in all injured wrists compared to the healthy wrist (*p* < 0.001, fractures with and without concomitant injuries vs. healthy wrist, Wilcoxon signed-rank test, *n* = 39; Figure 7A). 

The palmarflexion was also significantly reduced in patients suffering from distal radius fractures and arthroscopically addressed concomitant injuries compared to the healthy wrist (*p* < 0.001, fractures with concomitant injuries vs. healthy wrist, Wilcoxon signed-rank test, *n* = 30; Figure 7B). Patients with distal radius fracture and a TFCC lesion also showed a significant restriction of movement compared with the control (*p* = 0.01, fractures with lesions of the TFCC vs. healthy wrist, Wilcoxon signed-rank test, *n* = 20; Figure 7C). With regard to the palmarflexion, the follow-up examination showed no difference between distal radius fractures with isolated lesions of the SL ligament and the corresponding healthy wrist (*p* = 0.18). No significant difference was found between patients with distal radius fracture and combination injury from TFCC lesion and SL ligament lesion (*p* = 0.49) or between patients with distal radius fracture in combination with a TFCC lesion compared to distal radius fractures in combination with an SL ligament lesion (*p* = 0.3).

Patients suffering from distal radius fracture and arthroscopically addressed concomitant injuries showed no difference in palmarflexion compared to patients with isolated distal radius fractures (Figure 7D). 

## 4. Discussion

Our results demonstrate that arthroscopically supported repair of accompanying injuries of distal radius fractures, such as TFCC lesions, results in a comparable good function as isolated distal radius fractures. Hence, arthroscopy is an important tool for the treatment of intraarticular distal radius fractures being at risk for concomitant injuries. Therefore, our hypothesis was affirmed. 

In our study, 56 wrists were examined using a self-assessment questionnaire, the Munich Wrist Questionnaire [14]. We were able to examine 39 patients clinically for function and strength of the affected wrist. As a control, the healthy opposite side was used. In our study, 55 patients, including 56 arthroscopically assisted distal radius fractures, were enrolled within the study period from June 2012 to February 2015. This corresponds approximately to the numbers of the current literature. For example, Lindau et al. describe a follow-up of 43 patients [15] one year after arthroscopically assisted fracture treatment of a distal radius fracture in combination with a TFCC lesion. Gradl et al. included 18 patients with lesions of the SL ligament in a retrospective follow-up [16]. One of the few recent studies with a slightly larger number of patients is the prospective study by Kaspinova et al. [17], who enrolled 85 patients after arthroscopically assisted treated distal radius fracture. However, most studies investigate the occurrence of concomitant injuries. There are hardly any studies about the functional outcome after arthroscopic treatment of these. 

Of the 55 patients included in our study, 29 were female with a mean age of 52 ± 3 years, significantly older than the male patients, 41 ± 3 years of age. In a multicenter, retrospective cohort study by Pechlaner et al. [18], a total of 707 patients were enrolled to investigate the etiology and epidemiology of distal radius fractures. In this study, women aged 40–59 years were more often affected as well. The current literature review, therefore, suggests that the epidemiological data on age and gender in distal radius fractures with concomitant injuries are roughly equivalent to data from studies of distal radius fractures in general. This hypothesis is also underlined by the incidence of accompanying injuries in distal radius fractures. The data about accompanying injuries in distal radius fractures vary according to the literature [19]. We found an incidence of concomitant injuries in 70% of the patients studied. Of these, TFCC lesions were most frequent in 42%. In general, type C fractures according to AO classification were most frequently affected by concomitant injuries, which is in line with the indications for arthroscopic-assisted fracture treatment in distal radius fractures [1]. 

All 55 patients enrolled in our study were followed up using the Munich Wrist Questionnaire [14], a self-assessment score for analysis of the wrist function. The Munich Wrist Questionnaire is a validated questionnaire, published in 2016 by Beirer et al. [14]. 

It examines, as an extension of the common questionnaires, the subjective function of the wrist, the range of motion and the grip strength [14]. In addition, 39 patients in our study collective were clinically re-examined concerning mobility and strength in the affected wrist. The healthy wrist served as a control. 

In the self-assessment questionnaire follow-up, there was no functional difference in our study population between patients with arthroscopically treated concomitant injury and those with isolated distal radius fracture. We could not find any functional difference between fracture types or associated injuries after surgical fracture treatment. The fact that the patients with distal radius fracture and a concomitant injury had similar functional outcomes compared to those without accompanying injury, shows the effectiveness and importance of arthroscopic treatment of intraarticular distal radius fractures. 

The statistically missing functional difference between the different types of fractures may be due to the unequal distribution of our study population, with a majority of distal radius fractures type C to AO and hardly type A and few type B fractures. However, this, in turn, is due to the indication for arthroscopic fracture treatment, since, for example, the indication for type A fractures has only been made if an accompanying injury had already been detected by preoperative imaging.

We could not find a statistically significant functional difference between the patients with distal radius fracture and isolated TFCC lesion compared to those with isolated SL ligament lesion or combination injury from TFCC lesion with SL ligament lesion either. Of all the patients enrolled in our study, 39 patients were clinically re-examined for strength (forearms) and flexibility. As a control, the unaffected side was used. 

There was a statistically significant difference between affected and unaffected wrists in the clinical follow-up regarding the strength of the affected wrist. In comparison to the individual fracture types or accompanying injuries, a statistically significant difference could only be detected between fractures in combination with lesions of the SL ligament and healthy control. However, in the clinical examination of mobility, decreased dorsalextension and palmar flexion in the affected wrist were found in isolated distal radius fractures compared to distal radius fractures with TFCC lesions. In the presence of a distal radius fracture in combination with an isolated SL ligament rupture and combination injury from SL ligament rupture and TFCC lesion, no significant difference was found in comparison to the healthy opposite side. The importance of TFCC lesions in distal radius fractures is also shown in the study by Kasapinova et al. [20]. In a prospective study, patients with isolated distal radius fractures were compared to patients with distal radius fractures in combination with a TFCC lesion for their postoperative function in the affected wrist. The TFCC lesions were arthroscopically diagnosed but not addressed. Kaspinova et al. describes a significantly greater postoperative extent of pain in those patients with a combination injury, and also statistically significant inferior results in terms of function in the presence of a combination injury from distal radius fracture and TFCC lesion [20]. 

As our collective is older than 40 years and the TFCC lesions were central, as well as distal, some TFCC lesions might have a degenerative component as well. However, no radioulnar instability persisted after arthroscopic suture of the lesion, thus no open repair was needed. 

In our study, we found a statistically significant difference in postoperative strength but only a slight difference regarding postoperative mobility in patients with distal radius fractures in combination with isolated SL ligament rupture. This corresponds to the results of Gradl et al. In their retrospective study, they describe comparable functional results between patients with isolated distal radius fractures and distal radius fractures in combination with SL ligament injuries, if addressed intraoperatively [16]. Moreover, the consequences of a missed SL ligament tear with following SL dissociation and wrist arthrosis are fatal. Even if the SL ligament tear can be detected before carpal collapse has occurred, the results of reconstructive surgeries are often unsatisfactory. Arthroscopy is still the golden standard for the detection of an SL ligament tear [7]. 

The self-assessment results of our study show that arthroscopic treatment of concomitant injuries of distal radius fractures results in a similar wrist function compared to fractures without concomitant injuries. This leads to the conclusion that reconstruction of concomitant injuries in distal radius fractures is of great importance. For the detection of these injuries and treatment, an arthroscopic approach is needed. 

However, it remains still unclear if an arthroscopy should be performed mandatory in patients suffering from distal radius fractures and the decision is always an individual one discussed with the patient.

Moreover, arthroscopy is a time and money consuming procedure. Length of surgery is notably prolonged, more material is needed and, so far, no adequate financial compensation is determined. Therefore, patients should be selected carefully.

However, this study has several limitations. First, the number of wrists enrolled is small (56) although not too small compared with previously published studies.

Moreover, it is not a randomized controlled trial. Therefore, the control group existing of patients suffering from untreated concomitant lesions does not exist, as it would be unethical. 

In summary, in the presence of an intraarticular, distal radius fracture, it is highly likely that associated injuries such as TFCC lesions, SL ligament ruptures or combination injuries will occur. Accordingly, such fractures require a precise diagnosis and therapy adapted to the corresponding concomitant injury. In our study, we could show that there is little functional difference in the function of distal radius fractures in combination with concomitant injuries compared to isolated distal radius fractures, provided that the accompanying injuries are detected arthroscopically and addressed accordingly. In summary, our study demonstrates the high value of arthroscopy in the fracture management of distal radius fractures in combination with concomitant injuries in the restoration of wrist function. 

## Figures and Tables

**Table 1:** Fracture classification and concomitant injuries.

Characteristics of the Evaluated Patients	
AO fracture type A	5 (9%)
AO fracture type B	9 (16%)
AO fracture type C	42 (75%)
Distal radius fracture + isolated TFCC lesion	24 (43%)
Distal radius fracture + isolated SL ligament tear	8 (14%)
Distal radius fracture + TFCC lesion + SL ligament tear	7 (12.5%)

**Table 2:** Evaluation of the function using the MWQ.

Distal Radius Fracture + Concomitant Lesion	Distal Radius Fracture + Concomitant Lesion	Comparison of Function with Munich Wrist Questionnaire (MWQ)
Distal Radius Fracture + TFCC lesion (*n* = 24)	distal radius fracture + SL ligament tear (*n* = 8)	*t*-test (*p*-value = 1.0)
Distal Radius Fracture + TFCC lesion (*n* = 24)	distal radius fracture + TFCC lesion + SL ligament tear (*n* = 7)	*t*-test (*p*-value = 0.636)
Distal Radius Fracture + TFCC lesion (*n* = 24)	distal radius fracture without concomitant lesions (*n* = 17)	*t*-test (*p*-value = 0.979)
Distal Radius Fracture + SL Ligament tear (*n* = 8)	distal radius fracture + TFCC lesion + SL ligament tear (*n* = 7)	*t*-test (*p*-value = 0.779)
Distal Radius Fracture + SL Ligament tear (*n* = 8)	distal radius fracture without concomitant lesions (*n* = 17)	*t*-test (*p*-value = 0.954)
Distal Radius Fracture + TFCC lesion + SL Ligament tear (*n* = 7)	distal radius fracture without concomitant lesions (*n* = 17)	*t*-test (*p*-value = 0.775)
